# Territorial choruses of giant otter groups (*Pteronura brasiliensis*) encode information on group identity

**DOI:** 10.1371/journal.pone.0185733

**Published:** 2017-10-12

**Authors:** Christina A. S. Mumm, Mirjam Knörnschild

**Affiliations:** 1 Animal Behavior Lab, Institute for Biology, Freie Universität Berlin, Berlin, Germany; 2 Smithsonian Tropical Research Institute, Ancón, Panamáa; 3 Museum für Naturkunde, Leibniz Institute for Evolution and Biodiversity Science, Berlin, Germany; Texas A&M University College Station, UNITED STATES

## Abstract

Group living animals often engage in corporate territorial defence. Territorial group vocalizations can provide information about group identity, size and composition. Neighbouring groups may use this information to avoid unfavourable direct conflicts. Giant otters are highly social and territorial animals with an elaborate vocal repertoire. They produce long-range screams when they are alert or excited, i.e. in an alarm, isolation or begging context. Long-range screams are not only produced by one individual at a time (‘single screams’) but also by multiple group members simultaneously, resulting in a highly conspicuous ‘group chorus’. Wild giant otters regularly produce group choruses during interactions with predators, when they detect intruders in their territory or before group reunions after separation. Since single screams and especially group choruses probably contribute to the groups’ corporate territorial defence, we hypothesized that group identity is encoded in single screams and group choruses. We analysed vocalizations from five wild and three captive giant otter groups and found statistical evidence for a group signature in group choruses. Results for single screams were less conclusive, which might have been caused by the comparatively lower sample size. We suggest that giant otters may gain information on group identity by listening to group choruses. Group identity likely constitutes important social information for giant otters since territory boundaries of neighbouring groups can overlap and direct inter-group conflicts are severe. Therefore, group chorusing may contribute to the mutual avoidance of members from different groups.

## Introduction

One of the advantages of group living is corporate territorial defence [1–3]. To maintain a territory and to defend it against competitors, group members patrol territorial borders, signal their presence and ownership through marking, or actively fight against intruders [4–5]. Territorial marking can be done visually [6], chemically [7], or acoustically [8]. Acoustic group signals (often called ‘choruses’) can play an important role in territorial maintenance since loud, long-distance vocalizations allow animals to defend large breeding or foraging territories [9]. Group choruses might not only signal the presence of the resident group, but provide information on group composition, size, strength and willingness to fight [9]. Competitors, neighbours or potential intruders can use the information provided to assess the resource holding potential of the territory owners, and thereby the likeliness to win or lose a direct conflict [10–11]. Contests will only escalate when one competitor does not assess the full information about the other, or when the benefit of winning is much higher than the cost of being injured [10–11].

Choruses of different social groups may have distinct acoustic characteristics [12–14]. These between-group differences are most likely shaped by a combination of different factors. Geographical distance, genetic relatedness or isolation, environmental and morphological factors, as well as vocal convergence, vocal learning and cultural drift may all influence the development of acoustic differences between groups and similarities within groups [15–18]. Between-group differences can occur as exclusive group specific vocalizations, differences in the usage of certain vocalizations, or as distinct group signatures within certain vocalizations on a spatial scale [18].

In carnivores, vocal group chorusing finds its most prominent representatives in the howling of wolves [8], and the roaring of lions [19]. Several other social carnivores also engage in acoustic group signalling, e.g. African wild dogs [20], coyotes [21], hyenas [22], and giant otters [23–24]. Giant otters, *Pteronura brasiliensis*, are top predators in the Amazonian rainforests and wetlands [25]. They have a highly social organisation based on cooperation of group members [6]. Hunting and breeding success increase with group size, as larger groups show an increased rate of successful fish catches and provide more helpers to protect and raise the vulnerable cubs [26–27]. The social structure of this endangered otter species [28] is based on the ‘parent-brood model’ [29], with groups consisting of the reproductive alpha-couple and their offspring from two or three years [6,26,29]. However, groups can also contain unrelated individuals, indicating that the social structure is not restricted to an alpha pair and its offspring [29]. Changes in group structure result from dispersing subadults reaching sexual maturity [6, 29, 30–31], from the replacement of the alpha male or female [26,32] or from the incorporation of individuals after territorial fights [29]. Even though not all changes in group structure proceed peacefully [29], giant otters seem to predominantly avoid aggressive encounters, both within their group and with other giant otter groups within their home range [6,26,32–33]. Nevertheless, giant otters are highly territorial [26] and a group actively defends its territory [6]. This defence is especially important in areas where territories of neighbouring groups overlap [34]. Severe fights may occur between groups at territory borders [35], in areas with territorial overlap [36–37] or when a group tries to establish a new territory [37]. These conflicts may even result in the death of an otter [36]. Furthermore, infanticide by transient non-group members can occur [38]. In captivity, aggression occurs as a result of limited space, or between newly introduced animals ([39], own observations). These incidences of strong intraspecific aggression, directly linked to fitness costs, indicate that giant otters would benefit from effective ways to avoid such encounters. Giant otter groups mark their territory by olfactory and visual cues at distinct ‘scent-marking places’ [6,26,33]. Even though these places provide obvious information for neighbouring groups and possible intruders [40], they are stationary, and giant otters are highly mobile throughout the day [26]. Therefore, not only scent-marking but also acoustic communication, especially group choruses, may help to reduce aggressive conflicts in giant otters [41].

Giant otters have an elaborate vocal repertoire, comprising 19 to 22 distinct vocalization types with additional graded variants [23–24,42]. The vocal repertoire is shared among distant giant otter populations and, so far, no acoustic differences between social groups from the same population have been reported. The distinct vocalization types within the repertoire can be readily discriminated by acoustic parameters and the behavioural context involved [23–24]. When alarmed, e.g. when confronted with a predator or a conspecific intruder, giant otters produce several different vocalisation types such as snorts, whines and wavering screams [23–24]. Both whines and wavering screams are labelled ‘screams’ because of their shrill and ear-piercing acoustic properties and because of their roughness, i.e. their high rate of amplitude modulation.

Since screams are loud and low-frequency vocalizations that can be heard over large distances, they constitute long-range vocalizations [23–24]. Screams are not only produced by single giant otters but also by several group members simultaneously, resulting in a highly conspicuous group chorus [23–24]. In group choruses, screams from different individuals overlap and can be mingled with other vocalization types, like cohesion calls or aggressive growls [24,35]. Giant otters produce screams when they are highly aroused or alarmed, e.g. during aggressive interactions with caimans, after detecting intruders in their territory or when group members are separated and subsequently reunited with their group [23–25]. Screams are also produced while begging for food, especially in captive groups [23]. Numerous field observations suggest that single screams and group choruses constitute a form of territorial signalling [23–25]. Therefore, it is conceivable that single screams and group choruses encode information on group identity (i.e. a vocal group signature). Vocal signatures have been found in other vocalization types of giant otters, i.e. individual signatures in cohesion calls [43] and alarm snorts [44] or group signatures and sex-specific differences in alarm snorts [44].

In our present study, we analysed single screams (wavering screams and whines) and group choruses (consisting of overlapping wavering screams and whines from several individuals) to investigate acoustic group differences in giant otters. For single screams, we analysed whether individuals from the same group produced screams with group-specific acoustic parameters. For group choruses, we tested whether the vocal contribution of several group members created distinguishable acoustic differences between groups’ choruses (sensu [13]), since different groups were composed of different individuals. We hypothesized to find ‘group signatures’ in these vocalizations (between-group differences that allow the discrimination of different groups) since group signatures would facilitate corporate territorial defence.

## Materials and methods

### Ethical statement

Giant otters are an endangered species [28]. When working with wild giant otters in Peru, we obtained all necessary research permissions (No. 014 S/C- 2011-SERNANP-PNM, 014-2012-SERNANP-JEF, 017-2012-SERNANP-JEF and 0167-2012-DGFFS-DGEFFS) provided by SERNANP (Servicio Nacional de Áreas Naturales Protegidas), the Peruvian nature conservation authority and DGFFS (Dirección General Forestal y de Fauna Silvestre), the Peruvian agricultural department. Depending on the giant otters’ activities, we kept a minimum observing distance of 10–50 meters (and further increased the distance when new-born cubs were present).

When working with captive giant otters, we obtained research permissions from the respective persons in charge (Tierpark Hagenbeck: veterinarian, Zoo Dortmund: zoo director and Zoo Duisburg: curator). All zoos provided separable indoor and outdoor areas for giant otters. The indoor enclosures mainly served as retreat areas and were illuminated with artificial light. When a litter was born, public access to the retreat areas was prohibited. The outdoor enclosures, under natural light conditions, were always accessible for visitors. A daily amount of 2.0 to 6.0 kg of fish (trout, whiting and roach) was fed to the captive individuals. Additionally, they received fruit and vegetables for enrichment. All applicable international, national, and/or institutional guidelines for the care and use of animals were followed by the zoos.

### Study sites and study animals

We recorded five wild giant otter groups in Peru and three captive groups in German zoos (see S1 Table in the supporting information for details on group origin and composition). Group size varied from five to fifteen individuals, covering all age classes from new-born cubs to adults (giant otter age classes according to [39]). In 2011, we studied two wild groups within the Manu National Park at the lakes Cocha Cashu (N -11°53'3.9984'', E -71°24'28.0008'') and Cocha Salvador (N -11°59'45.9996'', E -71°13'59.0016”) from September to December. In 2012, we recorded two groups within the Tambopata National Reserve (Cocha Sandoval: N -12°36'29.4336”, E -69°2'26.9988”, Cocococha: N -12°49'0.624”, E -69°15'36.3456”) and one giant otter group in the reserve’s buffer zone (Cocha Tres Chimbadas: N -12°47'21.7932”, E -69°20'44.0988”) from April to July 2012. Giant otter groups in the German zoos Tierpark Hagenbeck, Zoo Duisburg and Zoo Dortmund were recorded in 2011.

### Recordings

We recorded the vocalizations with a directional microphone (Sennheiser, MKH 416-P48U3, frequency range: 40Hz-20kHz, sensitivity: (1kHz) 25mV/Pa ± 1dB) connected to a digital audio recorder (Zoom H2 Handy Recorder, 24 bit depth resolution, 96 kHz sampling rate) as wave files (48 or 96 kHz sampling rate, 24 bit depth resolution). To document the behavioural context, notes were spoken directly into the recordings after calling behaviour had ceased. Additionally, we filmed the behaviour during recording sessions (Sony DCR SR-35 camcorder). For this study, we defined three behavioural categories. The category ‘alarm’ included all situations in which giant otters engaged in highly alert behaviour, such as extreme monitoring or periscoping, fast back and forth swimming or rapidly patrolling the lakeshore. In the wild, alarm behaviour was mainly induced by caiman interactions. In captivity, alarm behaviour could be observed when a subadult giant otter took cubs from the den and carried them into the water. The category ‘begging’ referred to all situations in which giant otters begged for fish. In the wild, juveniles often begged group members for prey. In the zoos, all giant otters engaged in intense begging before feeding time. The category ‘isolation’ included all situations in which one or more giant otters were separated from their group (which only occurred in wild groups).

For the analysis of single screams, vocalizing individuals where identified by throat markings whenever possible. In Peru, this was done directly during observations, or subsequently when analysing the videos. In captive groups, individual identification was much easier due to decreased observing distance but, nevertheless, individual identification was not possible for all recordings. For the analysis of group choruses, however, it was not necessary to identify vocalizing individuals since we wanted to test whether overlapping vocalizations of multiple group members would create distinguishable acoustic differences between group choruses. Usually all group members participate in chorusing, which is why we are confident that we did not overestimate acoustic group differences based on the contributions of particularly vocal individuals in different groups.

Wild giant otters were observed and recorded by one observer per shift, following them in a one-person kayak. We covered the otters' daily activity period with four alternating three hours shifts from sunrise to sunset (around 5 am to 5 pm). In the zoos, one observer recorded giant otters from the outside of the enclosures. The daily activity period of the captive otters was covered by alternating three hour recording sessions in the morning, afternoon or evening.

### Selection and processing of the vocalizations

We classified the vocalizations according to the previously described vocal repertoire of giant otters [23–24]. We distinguished two types of scream vocalizations (‘single screams’) based on their acoustic properties, namely ‘wavering screams’ and ‘whines’ [23]; another study labelled both as ‘screams’ [24]. The original recordings were examined in Raven Pro (version 1.4, H. Mills et al., Ithaca, New York) and group choruses and single screams were selected whenever they had a good signal-to-noise-ratio and were not overlapped by interfering background noise. The selection by sound quality strongly reduced the number of vocalizations suitable for sound analysis. Background noise (easily detected as darkest parts in a grey-scale spectrogram) regularly overlapped with lower frequencies of group choruses and single screams, which could not be analysed accurately and were therefore discarded. From the original recordings, 50% of single screams and 65% of group choruses were selected for subsequent processing. Selected vocalizations were noise reduced (WavePad Sound Editor, version 4.52, NCH Software), and background noise not overlapping with the vocalization itself was erased manually (AviSoft SASlab Pro, version 5.1.23, R. Specht, Berlin, Germany). Then we normalized the amplitude of each vocalization to 100% of its maximum range, making it as loud as possible without changing its dynamic range (Raven Pro version 1.4, H. Mills et al., Ithaca, New York).

**Fig 1:**
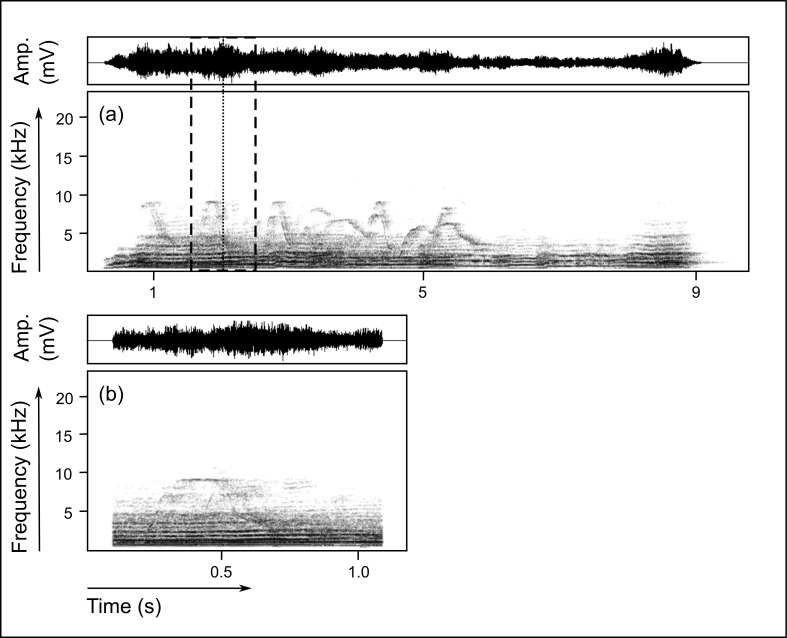
**Whole group chorus (a) and chorus excerpt of one second duration (b).** The group chorus was produced in an alarm context from the giant otters at Cocha Salvador (**a**). The dotted line indicates the location of the peak frequency, the dashed lines frame the excerpt of one second duration centred on the peak frequency. (**b**) Excerpt of the group chorus depicted in the upper panel. Spectrograms show frequency over time and were generated using a 1024-point FFT and a Hann window with 75% overlap. Oscillograms show amplitude as pressure changes over time. The audio file of the group chorus depicted in (**a**) is provided in the supporting information (S1 Audio).

To prepare single screams for automated acoustic parameter extraction, we cut each vocalization from its onset to its end and tapered 5 milliseconds of both onset and end. Then we added 100 milliseconds of silence before and after each scream. For group choruses, we selected a representative excerpt of one second duration for each chorus which was centred on the peak frequency of the whole chorus (Fig 1). Peak frequency was defined as the point of maximum power in a vocalization. The peak frequency’s time of occurrence in each group chorus was measured in Raven Pro (FFT: 512-point, window: Hann, overlap: 87.5%, temporal resolution: 0.667ms, DFT size: 512 samples, frequency resolution: 188 Hz). Subsequently, an excerpt of one second duration was extracted from the group chorus (0.5 seconds before and 0.5 seconds after the peak frequency of the whole chorus occurred; Fig 1). For the majority of group choruses, the peak frequency was located in the middle of the sequence. In rare cases where the peak frequency was very close to the beginning or end of a group chorus, we relocated the excerpt so that it still included the peak frequency but no silence. We tapered 5 milliseconds of onset and end of each excerpt and added 100 milliseconds of silence before and after each excerpt.

### Acoustic parameter extraction

Since single screams and group choruses are noisy signals with many nonlinearities (and, in the case of group choruses, consist of overlapping vocalizations), it was not feasible to measure ‘common’ acoustic parameters like the frequency contour or the amount of modulations. Instead, we applied a custom built MATLAB routine (using the speech processing toolbox ‘voicebox’ in MATLAB v. R2014a) to extract acoustic ‘features’ (mel-frequency cepstral coefficients; MFCCs) from single screams and chorus excerpts. MFCCs are spectral-based representations of entire signals, capturing most important features of signals in a compact form. This technique is widely used for human voice analysis and human speaker recognition (reviewed in [45]). Besides its use to identify individual speakers by voice cues [45], MFCCs have successfully been used for various other purposes such as music modelling [46], classification of emotion in speech [47], and the classification of livestock vocalizations [48]. MFCCs are based on the mel-scale which represents how human listeners perceive the distinction of voice pitch [45–46]. Because human pitch distinction is better for lower frequencies, the mel-scale is linear up to 1 kHz and logarithmic above, resulting in a stronger emphasis on low frequencies [45]. We chose the mel-scale because giant otter calls lie within the range of human speech and hearing; the vast majority of giant otter vocalizations are produced with peak frequencies below 4 kHz [23–24]. During the process of acoustic feature extraction, the information of the whole signal is condensed in several steps of calculations [49]. By applying a Hamming window function, the signal is first divided into overlapping frames of equal length and a Fourier transform (FFT) for each frame is computed. The resulting magnitudes (or powers) are subsequently mapped to the mel-scale and the spectrum is segmented into a number of critical bands by means of a filterbank, which consists of overlapping triangular filters. Subsequently, a discrete cosine transformation is applied to the logarithm of the filterband to calculate the MFCCs [46,49]. The feature extraction technique computed 27 MFCCs for chorus excerpts (N = 230) and single screams (N = 220) each. We used the 27 MFCCs in subsequent statistical analyses as we would use ‘normal’ acoustic parameters. All MFCC-data are provided in the supporting information (S2 Table).

### Statistics

To test for context-specific and group-specific differences in chorus excerpts and single screams, we performed discriminant function analyses (DFAs) which contained the extracted MFCCs as independent variables. To decide which of the 27 MFCCs to include in the DFAs, we divided each data set into a ‘trial’ set (1/3 of the total data) and ‘test’ set (2/3 of the total data) and conducted stepwise DFAs on each trial set. Stepwise DFAs automatically select acoustic parameters which are best suited for the respective discrimination task. The MFCCs selected in the stepwise DFAs were included simultaneously in subsequent DFAs on the test sets. Only results for the test sets are reported here; information on selected MFCCs can be found in the supporting information (S3 Table). For DFAs conducted on the test sets, we used cross-validation procedures to estimate the correct classification success (n−1 cross-validation procedure). DFAs were adjusted to the unequal number of analysed vocalizations per behavioural context or social group (Tables 1 and 2) by taking each vocalization’s probability to belong to a specific context or group into account (which is based on the total number of vocalizations per context or group). For each DFA, we report N_total_ (the total number of vocalizations in the trial set and the test set) and N_test_ (the number of vocalizations in the test set). In addition to the obtained cross-validated classification success, the expected random classification success (based on the number of contexts or groups to discriminate) was reported as well.

**Table 1:** Number of recorded group chorus excerpts and single screams (wavering screams / whines) for each social group in different behavioural contexts (alarm, begging, and isolation).

	Group chorus excerpts			Single screams (wavering screams/whines)		
Social groups	Alarm	Begging	Isolation	Alarm	Begging	Isolation
Cocha Cashu	39	-	2	13 (1/12)	5 (3/2)	-
Cocococha	5	-	-	-	-	-
Cocha Salvador	15	-	10	9 (3/6)	8 (5/3)	3 (3/0)
Cocha Sandoval	13	-	1	7 (5/2)	11 (1/10)	3 (1/2)
Cocha Tres Chimbadas	-	5	2	-	45 (26/19)	8 (8/0)
Zoo Dortmund	-	32	N/A	-	18 (18/0)	N/A
Zoo Duisburg	20	29	N/A	9 (8/1)	14 (13/1)	N/A
Zoo Hagenbeck	7	50	N/A	-	47 (40/7)	N/A
*Total*	*99*	*116*	*15*	*38 (17/21)*	*148 (106/42)*	*14 (12/2)*

For single screams, numbers of wavering screams and whines are given in parentheses. The isolation context did not exist for captive groups because group members were never separated in zoos.

**Table 2:** Number of recorded single screams for each social group in a begging context.

Social groups	Wavering screams	Whines
Cocha Cashu	(3)	(2)
Cocha Salvador	5	(3)
Cocha Sandoval	(1)	10
Cocha Tres Chimbadas	26	19
Zoo Dortmund	(18)	-
Zoo Duisburg	13	(1)
Zoo Hagenbeck	40	7
*Total included in DFA*	*84*	*36*
*Total*	*106*	*42*

Groups with numbers in parentheses were not included in the analyses due to low sample sizes (N<5 per group) or because all recorded vocalizations stemmed from the same individual (Zoo Dortmund).

We analysed 230 group chorus excerpts from eight giant otter groups. We first tested whether the extracted MFCCs contain sufficient information to distinguish between chorus excerpts produced in different behavioural contexts (alarm: N = 99, begging: N = 116, isolation: N = 15; N_total_ = 230, N_test_ = 153). Subsequently, we performed DFAs within behavioural contexts to test whether chorus excerpts could be correctly assigned to the respective groups. We used chorus excerpts from six groups for the category alarm (5–39 excerpts per group; N_total_ = 99, N_test_ = 66) and chorus excerpts from four groups for the category begging (5–50 excerpts per group; N_total_ = 116, N_test_ = 77). We did not have enough chorus excerpts for the category isolation (4 groups, 1–10 excerpts per group; N_total_ = 15) to perform statistical tests. The begging context was only rarely observed in wild groups, the isolation context was never observed in captive groups (Table 1).

We analysed 200 single screams, 135 wavering screams from seven groups and 65 whines from six groups. Again, we first tested whether the extracted MFCCs contain sufficient information to distinguish between single screams produced in different behavioural contexts. For wavering screams, we tested the distinction between all three behavioural contexts (alarm: N = 17, begging: N = 106, isolation: N = 12; N_total_ = 135, N_test_ = 90); for whines, we tested only the distinction between alarm and begging (alarm: N = 21, begging: N = 42, isolation: N = 2; N_total_ = 63, N_test_ = 42). Subsequently, we performed additional DFAs within the behavioural context begging (for which we had the largest sample size; 84 wavering screams, 36 whines; Table 2) to test whether wavering screams or whines could be correctly assigned to the respective groups. One group (Zoo Dortmund) had to be excluded from this analysis because all wavering screams and whines recorded there stemmed from only one particularly heavily begging individual (a situation that would have confounded individual and group signatures). We conducted a DFA with wavering screams from four groups (5–40 wavering screams per group; N_total_ = 84, N_test_ = 65) and another DFA with whines from three groups (7–19 whines per group; N_total_ = 36, N_test_ = 24). Our data did not allow for a nested design (individuals nested within groups) because not all single screams could be reliably associated to a specific individual (we were able to assign 46%, i.e. 92 of 200 single screams). However, it was very obvious when one individual was much more vocal than its group members (e.g. in the Zoo Dortmund group which was excluded from analyses on group differences), which makes us confident that we recorded different individuals in each group (even though individual identification of a particular screaming giant otter was not always possible). All statistical tests were performed with SPSS (version 21, SPSS Inc., Chicago, IL, U.S.A.).

## Results

**Fig 2:**
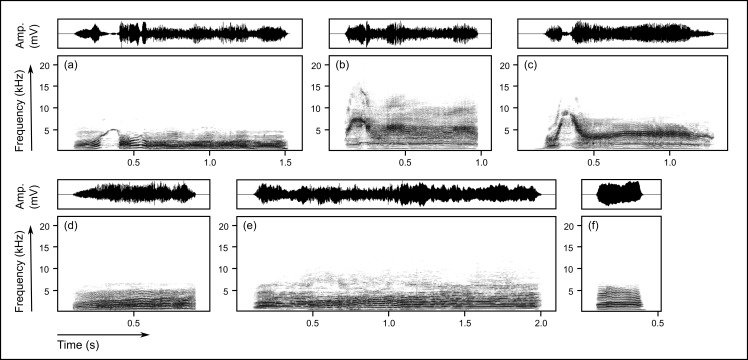
Single screams (wavering screams and whines) produced in different behavioural contexts. Upper panel: wavering screams produced in (**a**) alarm (Cocha Sandoval), (**b**) begging (Zoo Duisburg) and (**c**) isolation (Cocha Tres Chimbadas). Lower panel: whines produced in (**d**) alarm (Cocha Cashu), (**e**) begging (Zoo Tierpark Hagenbeck) and (**f**) isolation (Cocha Sandoval). Spectrograms show frequency over time and were generated using a 1024-point FFT and a Hann window with 75% overlap. Oscillograms show amplitude as pressure changes over time. The audio files of the vocalizations depicted in (**a-f**) are provided in the supporting information (S2–S7 Audios).

Wild groups mainly produced group choruses (Fig 1) and single screams (Fig 2) in an alarm or isolation context, whereas the captive groups used group choruses and single screams most frequently in a begging context (Table 1). All captive groups but only one wild group emitted group choruses for begging (the wild group chorused during a severe intra-group conflict over captured prey). For single screams, the differences between call usage in begging wild and captive groups was less pronounced, but captive groups were generally more likely to engage in begging behaviour than wild groups. The alarm context, frequently observed in wild groups when predators or territorial intruders were encountered, occurred only once in one captive group (when a subadult giant otter removed cubs from the den and carried them into the water). The isolation context did not exist for captive groups because they were never separated.

### Group choruses

**Fig 3:**
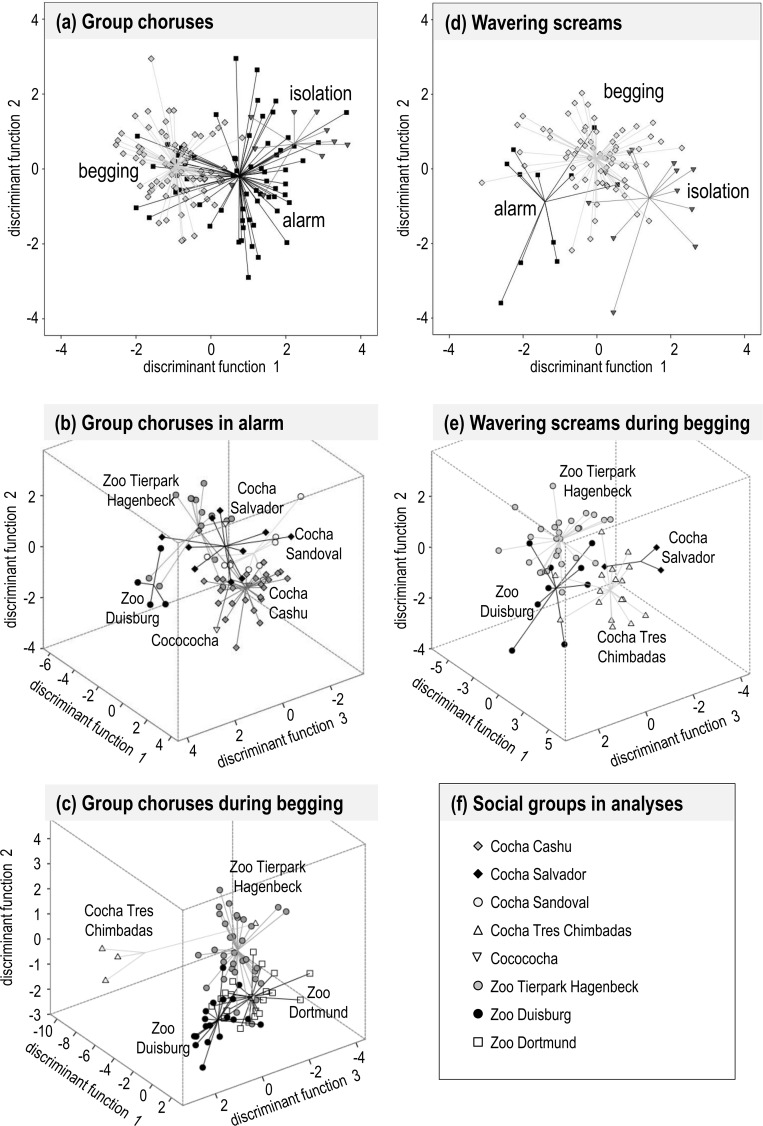
**Signal spaces depicting context-specific differences and group signatures for group choruses (a-c) and wavering screams (d-e).** The signal spaces are defined by discriminant function analyses (on test data sets). Relative positions of vocalizations are marked with different symbols; lines depict the connection between vocalizations from the same behavioural context (**a, d**) or social group (**b, c, e**) to their respective centroids. Group chorus excerpts from three behavioral contexts (**a**), from six giant otter groups in alarm (**b**) and from four giant otter groups during begging (**c**) are shown. Wavering screams from three behavioral contexts (**d**) and from four giant otter groups during begging (**e**) are shown. Symbols of all social groups in analyses are listed in (**f**).

Group chorus excerpts encoded sufficient information to be correctly assigned to the respective behavioural context in which they were produced (alarm, begging or isolation; Fig 3A). A DFA (8 MFCCs, N_total_ = 230, N_test_ = 153) classified 71.9% of chorus excerpts to the correct behavioural context (3 contexts; random classification success: 33.33%). Within behavioural contexts, group chorus excerpts could be correctly assigned to the social group which produced them. Within the alarm context, a DFA (4 MFCCs, N_total_ = 99, N_test_ = 66; Fig 3B) classified 68.2% of chorus excerpts to the correct social group (6 groups; random classification success: 16.67%). Within the begging context, a DFA (4 MFCCs, N_total_ = 116, N_test_ = 77; Fig 3C) classified 76.6% of chorus excerpts to the correct social group (4 groups; random classification success: 25%). Details on assessment of model fit are provided in the supporting information (S3 Table). Within the behavioral context isolation, we could not test for group differences because sample sizes were too low (N = 15; Table 1).

### Single screams

Single screams, i.e. wavering screams and whines, encoded sufficient information to be correctly assigned to the behavioural context in which they were produced. A DFA (5 MFCCs, N_total_ = 135, N_test_ = 90; Fig 3D) classified 78.9% of wavering screams to the correct behavioural context (alarm vs. begging vs. isolation; random classification success: 33.3%). A second DFA (2 MFCCs, N_total_ = 63, N_test_ = 42) classified 71.4% of whines to the correct behavioural context (alarm vs. begging; random classification success: 50%). Within the behavioural context begging (for which we had the largest sample size), wavering screams and whines could be assigned to the social group which produced them but the results were less conclusive than for the context discrimination. A DFA (7 MFCCs, N_total_ = 84, N_test_ = 56; Fig 3E) classified 82.1% of wavering screams to the correct social group (4 groups; random classification success: 25%). A second DFA (1 MFCC, N_total_ = 36, N_test_ = 24) classified 66.7% of whines to the correct social group (3 groups; random classification success: 33.33%).

In general, the classification results were much stronger for group chorus excerpts than for single screams. Within single screams, evidence for a context- and group-specific classification was more convincing for wavering screams than for whines (S3 Table in supporting information).

## Discussion

Group choruses and single screams (wavering screams and whines) encoded information on the behavioral context in which they were produced. Moreover, group choruses encoded a group signature, i.e. sufficient information to discriminate between different social groups. The statistical evidence for a group signature in single screams was less conclusive. This difference might be caused by the comparatively lower sample size for single screams; however, it is also possible that group identity is not encoded in all types of giant otters’ long-range vocalizations.

Wild giant otter groups were recorded in five lakes which were located in similar habitat, namely lowland tropical rainforest with a distinct temperature variation and a pronounced dry season [26], which is why we do not believe that vocal differences between groups were caused by differences in the respective environment. Correspondingly, a study on alarm calls (so called ‘snorts’) of giant otters in Brazil did not find noteworthy vocal differences between groups which were less than 100 km apart [44]. A strong influence of environmental and/or genetic factors on vocal group differences is more likely to be found on a larger geographical scale (i.e. on the population level). A comparison between vocalizations from giant otter groups living in distant and more diverse habitats like the Peruvian lowland forest, the Brazilian Pantanal with flooded forest [50], or the Brazilian Cantão region on the borders of three different biomes [51] might clarify which factors influence vocal differences between giant otter groups. The difference in usage frequency of group choruses and single screams which we found in the present study most likely represents a discrepancy in living conditions between wild and captive giant otter groups and should therefore not be interpreted as a true group difference.

Playback experiments are needed to test whether giant otters’ can discriminate between different social groups solely based on acoustic information encoded in group choruses or single screams. Own preliminary playback data from groups in German zoos indicates that a forced-choice paradigm is not well-suited for giant otters, at least not in captivity. Instead we suggest using a habituation-dishabituation paradigm which has been successfully applied in playback experiments with captive giant otters before [43]; this paradigm may be better suited for investigating whether giant otters can perceive the acoustic differences in group choruses or single screams.

Wild giant otters produce group choruses and single screams in alarm and isolation contexts or when they are begging for prey items. The specific social functions of these vocalizations can be subdivided into intra-group and extra-group functions. A clear intra-group function of single screams and group choruses can be observed in the isolation context (and the subsequent reunion of group members). Giant otter groups are highly mobile; they often split up into smaller subgroups during their activity period [26] and reunite later. Inexperienced juveniles often get isolated during fishing and are then exposed to predators ([52], own observations). In both scenarios, single screams and group choruses can help giant otters to reunite with their group members. Long-range vocalizations facilitate group reunions in many highly mobile species as they offer the opportunity to localize group members and communicate with them over large distances [9,53–56]. In giant otters, exchanging wavering screams or listening to the group chorus could not only inform isolated giant otters about the current location of a particular social group, but also whether it is their own social group or not. Multiple intra-group functions of group choruses like coordination, reunion and location signalling have also been described for other social carnivores (e.g., lions [57], hyenas [22], African wild dogs [20], and wolves [58–60]).

A clear extra-group function of single screams and group choruses can be observed in the alarm context, when giant otters encounter predators or unfamiliar conspecifics intruding into their territory. While predators like caiman and jaguars might be actively mobbed [61] with the help of ear-piercing screams and group choruses, these conspicuous vocalizations can also help giant otters to avoid direct encounters with foreign and potentially hostile conspecifics [6,25,33,35]. Correspondingly, the functions of vocal group signatures in other group-living territorial animals are also related to territorial maintenance and resource defence (e.g., lions [57], hyenas [22], African wild dogs [20], coyotes [21], and wolves [58–59]). Group chorusing may encode group identity (wolves [14], laughing kookaburras [12], green woodhoopoes [13]; but see Australian magpies [62]) and signal group size (lions [63], red howler monkeys [64], grreen woodhoopoes [65]; but see wolves [66]). Wolves and lions use their group calls to actively avoid agonistic encounters [57,59], whereas hyenas and African wild dogs also emit choruses during confrontations with other groups [20,22]. Another extra-group function of single screams and group choruses is related to the occurrence of infanticide, which is not uncommon in otters in general [38,40]. For giant otters, the ‘babysitter system’ (one of the elder group members stays with the cubs to guard them) is of crucial importance to avoid infanticide [26]. However, babysitting does not occur in all giant otter groups [30] and smaller groups might not have the numbers to always leave a babysitter with the vulnerable cubs. Therefore, it should be advantageous for giant otters to signal their presence and willingness to fight to repel infanticidal intruders from their territory. Correspondingly, hyenas increase whooping close to their den to repel potentially infanticidal non-group members [22].

We cannot prove unambiguously that giant otters actively use single screams and group choruses for territorial defence (in addition to their function as alarm, cohesion and begging calls), but we consider it to be most likely, since single screams and group choruses are highly conspicuous long-range signals. However, even if single screams and group choruses were not directed at non-group members at all (thus having only intra-group functions), competitors could nevertheless eavesdrop on them and use the encoded information on group identity.

In conclusion, we show that group choruses and single screams of giant otters encode group signatures that could be used to discriminate between different groups. Since group choruses and single screams are loud vocalizations that can be heard over a distance of several hundred meters, vocal group signatures may facilitate inter-group spacing and help to avoid severe between-group conflicts, especially between resident group members and transient intruders.

## Supporting information

S1 Table Origin and composition of giant otter groups in our study. (XLSX)

S2 Table MFCC-data for group choruses, wavering screams and whines. (XLSX)

S3 Table Assessment of model fit and confusion matrices of the discriminant function analyses conducted on group chorus excerpts and single screams. (XLSX)

S1 Audio Group chorus in an alarm context (Cocha Salvador). Spectrogram provided in Fig 1A.
(WAV)

S2 Audio Wavering scream in an alarm context (Cocha Sandoval). Spectrogram provided in Fig 2A.
(WAV)

S3 Audio Wavering scream in a begging context (Zoo Duisburg). Spectrogram provided in Fig 2B.
(WAV)

S4 Audio Wavering scream in an isolation context (Cocha Tres Chimbadas). Spectrogram provided in Fig 2C.
(WAV)

S5 Audio Whine in an alarm context (Cocha Cashu). Spectrogram provided in Fig 2D.
(WAV)

S6 Audio Whine in a begging context (Zoo Tierpark Hagenbeck). Spectrogram provided in Fig 2E.
(WAV)

S7 Audio Whine in an isolation context (Cocha Sandoval). Spectrogram provided in Fig 2F.
(WAV)
